# Protein Misfolding, Signaling Abnormalities and Altered Fast Axonal Transport: Implications for Alzheimer and Prion Diseases

**DOI:** 10.3389/fncel.2019.00350

**Published:** 2019-07-30

**Authors:** Emiliano Zamponi, Gustavo F. Pigino

**Affiliations:** ^1^Department of Molecular, Cellular, and Developmental Biology, University of Colorado Boulder, Boulder, CO, United States; ^2^Laboratorio de Neuropatología Experimental, Instituto de Investigación Médica Mercedes y Martín Ferreyra, INIMEC-CONICET-Universidad Nacional de Córdoba, Córdoba, Argentina

**Keywords:** fast axonal transport, kinesin-1, casein kinase 2, signaling, synaptic dysfuction, protein misfolding, prion protein

## Abstract

Histopathological studies revealed that progressive neuropathies including Alzheimer, and Prion diseases among others, include accumulations of misfolded proteins intracellularly, extracellularly, or both. Experimental evidence suggests that among the accumulated misfolded proteins, small soluble oligomeric conformers represent the most neurotoxic species. Concomitant phenomena shared by different protein misfolding diseases includes alterations in phosphorylation-based signaling pathways synaptic dysfunction, and axonal pathology, but mechanisms linking these pathogenic features to aggregated neuropathogenic proteins remain unknown. Relevant to this issue, results from recent work revealed inhibition of fast axonal transport (AT) as a novel toxic effect elicited by oligomeric forms of amyloid beta and cellular prion protein PrP^C^, signature pathological proteins associated with Alzheimer and Prion diseases, respectively. Interestingly, the toxic effect of these oligomers was fully prevented by pharmacological inhibitors of casein kinase 2 (CK2), a remarkable discovery with major implications for the development of pharmacological target-driven therapeutic intervention for Alzheimer and Prion diseases.

## Introduction

Adult-onset misfolding diseases are among the most challenging disorders faced by modern molecular medicine. A pathogenic feature common to these diseases includes the accumulation of aggregated proteinaceous entities. By mid 20th century, it became clear that protein aggregates were the culprit of misfolding diseases, including Alzheimer’s (AD) and prion diseases (PrDs) (Hardy and Selkoe, 2002; Colby and Prusiner, 2011). More recently, the “amyloid cascade hypothesis” was proposed, suggesting that a cascade of pathological events associated with extracellular accumulation of amyloid precursor protein (APP) fragments underlies AD (Hardy and Higgins, 1992). This hypothesis was later extended to other misfolding diseases, leading to a modified hypothesis that included the notion that smaller, oligomeric intraneuronal aggregates may play a more relevant pathological role in AD and PrDs, (Takahashi et al., 2002; Forloni et al., 2016; Cline et al., 2018; Ono, 2018). As these disorders associated with small soluble aggregates (oligomers ranging from 4 to 200 kDa), the term “oligomeropathies” was coined to emphasize this notion (Forloni et al., 2016). Still, after many decades of intense research, mechanisms linking oligomeric protein aggregates to disease pathogenesis remain elusive.

Synaptic disfunction and axonal pathology are common early pathological features shared by neurons affected in protein misfolding diseases (Selkoe, 2002; Senatore et al., 2013; Chiesa, 2015; Soto and Pritzkow, 2018), suggesting that pathological misfolded proteins, including amyloid beta (Aβ) and PrP^C^, may alter cellular processes critical for synaptic and axonal function. One such process involves fast axonal transport (AT) a cellular process crucial for homeostatic maintenance of pre and postsynaptic compartments underlying functional neuronal connectivity (Ermolayev et al., 2009a,b; Pigino et al., 2009; Zamponi et al., 2017). In this mini-review, we discuss how two unrelated pathogenic proteins bearing oligomeric conformation, amyloid beta (oAβ-42) and cellular prion protein (oPrP^C^), inhibit AT by altering a common signaling pathways important for AT regulation. Implications of these in the Conclusion section.

## Early Synaptic Dysfunction and Neuritic Pathology in Protein Misfolding Diseases

Decades of research revealed neuronal synaptic terminals as primary targets in many protein misfolded diseases (Jeffrey et al., 2000; Selkoe, 2002; Conforti et al., 2007; Siskova et al., 2009). Accordingly, recent studies determined that abnormally folded tau and oligomeric amyloid beta, hallmarks AD proteins, inhibit synaptic transmission through a mechanism involving aberrant activation of the proteins kinases GSK3β and CK2, respectively (Moreno et al., 2009, 2016). Early synaptic dysfunction and axonal pathology represent common pathological events to all these disorders, preceding months or even years before any signs of overt neuronal cell death (Moreno and Mallucci, 2010; Adalbert and Coleman, 2013). Cumulative evidence indicates that deficits in neuronal connectivity associated with synaptic disfunction and axonal degeneration, rather than the loss of specific population of vulnerable neurons, underlies the clinical manifestation of each disease (Chiesa et al., 2005; Brady and Morfini, 2010; Coleman, 2011). Accordingly, therapeutic strategies based on preventing neuronal apoptosis failed to alter the progression of clinical symptoms in different animal models of protein misfolding diseases, including PrDs, amyotrophic lateral sclerosis (ALS) and PD (Chiesa et al., 2005; Gould et al., 2006; Waldmeier et al., 2006). Remarkably, eliminating cellular prion protein on mice infected with prions that normally develop the classic prion pathology and clinical signs of neurodegeneration (Mallucci et al., 2007; White et al., 2008) recovered synaptic dysfunction, which further prevented neuronal loss (Moreno and Mallucci, 2010). Therefore, the available information strongly suggests that preserving neuronal connectivity may represent an effective therapeutic strategy (Lingor et al., 2012). However, the development of such approaches requires the knowledge of pathogenic mechanism underlying loss of neuritic connectivity in all these unrelated neurological disorders (Luo and O’Leary, 2005; Conforti et al., 2007; Gerdts et al., 2016).

The development of mouse models for misfolding disorders was a major breakthrough that allowed an evaluation of hypothesis-driven disease mechanisms (Suter and Scherer, 2003). However, a major obstacle has been the scarcity of appropriate experimental systems that allow a direct evaluation of aggregation-dependent effects of neuropathogenic proteins. Within this context, the isolated squid axoplasm and squid giant synapse preparation represents unique experimental systems (Song et al., 2016). Isolated squid giant axon is independent of any nuclear or synaptic activity contributions, which cannot be achieved when working with mammalian neurons either *in vitro* or *in vivo* (Grant et al., 2006; Kanaan et al., 2012; Song et al., 2016). One putative limitation for the squid giant axon, as well as other invertebrate model such as *Drosophila melanogaster* and *Caenorhabditis elegans*, could be the state of conservation on regulatory mechanisms for AT between mammalian and invertebrate neurons. In this regard, we and others have shown that every specific axonal activity explored in the squid *Loligo pealeii* is conserved from cephalopods to humans. The *Loligo pealeii* was a pioneering animal model that provided fundamental insights into nerve cell excitability (Schwiening, 2012). Furthermore, it was instrumental for the discovery of kinesin-1 (Brady, 1985; Vale et al., 1985) and its regulatory mechanisms (Brady and Morfini, 2017), as well as the determination of the specific molecular mechanisms involved in synaptic transmission (Llinas et al., 1980).

## Oligomeric Forms of Aβ-42 and PrP^C^ Promote Aberrant Activation of the Protein Kinases Gsk3β and Ck2

A common pathological feature displayed by many adult onset aggregopathies is aberrant patterns of protein phosphorylation, which indirectly reflects alterations in the activity of phosphotransferases (Walaas and Greengard, 1991; Baskaran and Velmurugan, 2018). Cytoskeletal components of the axonal compartment, including the microtubule-associated protein tau and neurofilaments, are the most widely reported neuronal proteins aberrantly phosphorylated in AD and PrDs (Stoothoff and Johnson, 2005; Holmgren et al., 2012; Rudrabhatla, 2014).

In the last two decades of pharmacological research working with multiple cellular and animal models, it has become clear that GSK3-β kinase plays a key role in AD and PrDs pathology (Llorens-Martin et al., 2014). Significantly, GSK3β activity has been shown to be abnormally activated by the AD associated oligomeric Aβ-42 peptide (oAβ-42) and by PrP (Perez et al., 2003; Pigino et al., 2009; Decker et al., 2010; Tang et al., 2012; Simon et al., 2014). In addition, extracellular fibrillar Aβ-42 (fAβ) and either extracellular or intracellular oAβ-42 were found to activate CK2 both *in vivo* and *in vitro* (Chauhan et al., 1993; De Felice et al., 2009; Pigino et al., 2009; Tang et al., 2012; Ramser et al., 2013). Making this even more compelling, PrP reportedly associates with and activates CK2 (Meggio et al., 2000; Chen et al., 2008; Zamponi et al., 2017). Together these experimental evidences strongly indicates that oAβ-42 and oPrP promote activation of neuronal GSK3β and CK2 kinases (Pigino et al., 2009; Zamponi et al., 2017), a discovery bearing major implications for both AD and PrP pathogenesis.

Since most kinases have many different neuronal substrates, they could potentially affect a wide variety of cellular processes, including gene transcription (Whitmarsh, 2007; Thapar and Denmon, 2013; Gao and Roux, 2015), cytoskeleton organization (Rudrabhatla, 2014), protein degradation and mitochondrial function, among others. However, the precise molecular events linking these processes to synaptic dysfunction and axonal pathology have yet to be discovered. On the other hand, we do know AT is a process of utmost importance for maintaining normal axonal and synaptic function (Gibbs et al., 2015; Zamponi et al., 2017). In support, loss of function mutations in specific subunits of kinesin-1 and cytoplasmic dynein, major motor proteins responsible for the execution of AT, cause neuropathologies featuring synaptic dysfunction and axonal pathology early in the course of disease (Reid, 2003; Brady and Morfini, 2010).

## Fast Axonal Transport Alterations in Alzheimer and Prion Diseases

**FIGURE 1:**
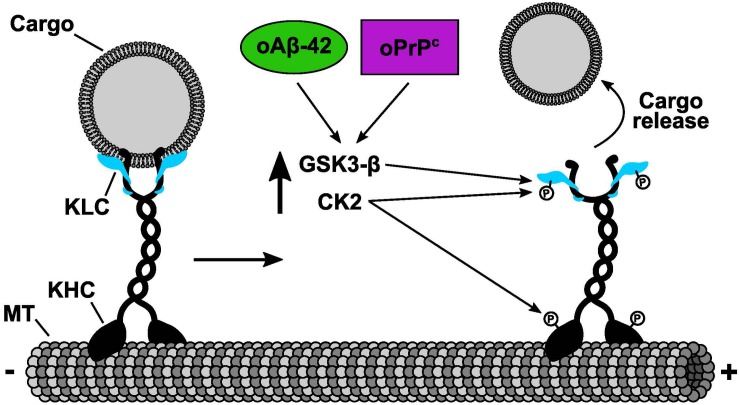
Common molecular mechanism of fast axonal transport inhibition shared by oPrP^C^ and oAβ-42. Cellular and pharmacological data determined that both oPrP^C^ and oAβ-42 induced fast axonal transport inhibition. The inhibitory mechanism was mediated by the activation of endogenous CK2 and GSK3β that in turn phosphorylated KLCs. Phosphorylation of KLCs (letter P on KLCs) promoted the detachment of conventional kinesin from its transported vesicular cargoes. We and others have shown that CK2 can also phosphorylate kinesin-1 heavy chains (KHCs) (letter P on KHCs). Based on our previous results working with KHCs phosphorylation, we predict an additional mechanism of fast axonal transport inhibition induced by CK2 phosphorylation on KHCs which in turn will promote a reduction of kinesin-1 association to microtubules (MTs).

In the last decade, genetic evidences have shown that alterations in kinesin and cytoplasmic dynein motor functions underlie a group of neuropathies (Brady and Morfini, 2010, 2017). Interestingly, all of these disorders display synaptic dysfunction and l axonopathy, signature pathogenic events associated with dying-back degeneration of neurons (Brady and Morfini, 2010). Although these neuropathies are associated with functional mutations in molecular motors, it became apparent that many more adult onset aggregopathies present defects in AT, including AD, and PrDs (Gibbs et al., 2015; Brady and Morfini, 2017; Zamponi et al., 2017). However, AT failure in these neuropathies was a result of alterations in phosphotransferase activities that regulate kinesin and dynein motor functions, rather than through mutation-based loss of motor activities (Brady and Morfini, 2017). Our recent results showed that cellular PrP can activate endogenous axonal CK2 activity and induce a dramatic inhibit AT of various membrane-bound organelles including synaptic vesicles and mitochondria (Zamponi et al., 2017). Abnormally activated CK2 in turn phosphorylates light chains subunits of kinesin-1, inducing a dissociation of this motor protein with its transported cargoes (Figure 1). Consistent with this molecular mechanism, inhibition of endogenous CK2 activity by specific pharmacological CK2 inhibitors prevented oPrP-induced AT inhibition in both isolated squid axoplasm and mammalian neurons (Zamponi et al., 2017). Remarkably, we and others have shown previously the same mechanism of AT inhibition induced by the AD related peptide oAβ-42 (Pigino et al., 2009; Tang et al., 2012). These important discoveries represent a message of hope for the development of therapies to treat aggregopathies involving compromised AT. In particular, these insights would be crucial for treating disorders that are induced by aggregated misfolded proteins capable of altering phosphotransferases important for regulating AT, a vitally important neuronal process that sustain normal axon functions and synaptic activities.

## Conclusion

For many decades the research on pathological mechanisms associated to adult onset neurological disorders such as AD and other aggregopathies, was focused almost exclusively on preventing neuronal cell death. The development of animal models, specifically focusing on these devastating diseases, has helped in the formulation of new hypothesis driven pathological mechanisms. Many research programs have developed effective ways of preserving neurons affected in these animal models, however, little or no progress was achieved in stopping or slowing the progression of these diseases. Indeed, cardinal research programs aimed to genetically prove the direct involvement of apoptosis, a pathological component of AD, PD, PrD, and ALS, determined that although apoptosis plays an important role in these diseases, preserving affected neurons did not prevent the clinical symptoms or synaptic dysfunction and loss (Chiesa et al., 2005; Gould et al., 2006).

The discovery that functional mutations in kinesin-1 and cytoplasmic dynein, the main molecular motors responsible for neuronal AT, suffice to promote dying back neuropathies was a major step forward (Brady and Morfini, 2010). However, mutations in molecular motors are rare, usually embryonic lethal, and only account for a small proportion of neurological disorders. It has become established in recent years, that deficiencies in AT are associated to a larger group of misfolding diseases including AD, PD, and PrDs. And, current research indicates that AT deficiencies observed in these diseases are induced by altered kinases involved in regulation of AT (Gibbs et al., 2015; Brady and Morfini, 2017). Altogether, this molecular and pharmacological information will set the basis for developing novel target-driven pharmacological interventions specific for each disease. These interventions will in turn ameliorate neuronal AT and therefore prevent or slow down the dying back progression of axonal degeneration and loss (Brady and Morfini, 2017).

## Author Contributions

GP wrote the original manuscript. EZ designed and draw the figure.

## Conflict of Interest Statement

The authors declare that the research was conducted in the absence of any commercial or financial relationships that could be construed as a potential conflict of interest. The reviewer, GM, declared a past co-authorship with one of the authors, GP, to the handling Editor.
